# A case–control study of the causes of acute respiratory infection among hospitalized patients in Northeastern Laos

**DOI:** 10.1038/s41598-022-04816-9

**Published:** 2022-01-18

**Authors:** Koukeo Phommasone, Xaipasong Xaiyaphet, Jose A. Garcia-Rivera, Robert D. Hontz, Viengmone Pathavongsa, Patsalin Keomoukda, Malavanh Vongsouvath, Mayfong Mayxay, Manivanh Vongsouvath, Paul N. Newton, Elizabeth A. Ashley, Audrey Dubot-Pérès

**Affiliations:** 1 Lao-Oxford-Mahosot Hospital-Wellcome Trust Research Unit, Microbiology Laboratory, Mahosot Hospital, Vientiane, Lao PDR; 2 U.S. Naval Medical Research Unit TWO (NAMRU-2), Singapore and Phnom Penh, Cambodia; 3 Xiengkhuang Provincial Hospital, Phonsavan District, Xiengkhuang Province Lao PDR; 4 Institute of Research and Education Development (IRED), Ministry of Health, Vientiane, Lao PDR; 5 University of Health Sciences, Ministry of Health, Vientiane, Lao PDR; 6 Centre for Tropical Medicine and Global Health, Nuffield Department of Medicine, University of Oxford, Oxford, UK; 7 Unité des Virus Émergents (UVE: Aix-Marseille Univ-IRD 190-Inserm 1207), Marseille, France

**Keywords:** Microbiology, Medical research

## Abstract

With the advent of highly sensitive real-time PCR, multiple pathogens have been identified from nasopharyngeal swabs of patients with acute respiratory infections (ARIs). However, the detection of microorganisms in the upper respiratory tract does not necessarily indicate disease causation. We conducted a matched case–control study, nested within a broader fever aetiology project, to facilitate determination of the aetiology of ARIs in hospitalised patients in Northeastern Laos. Consenting febrile patients of any age admitted to Xiengkhuang Provincial Hospital were included if they met the inclusion criteria for ARI presentation (at least one of the following: cough, rhinorrhoea, nasal congestion, sore throat, difficulty breathing, and/or abnormal chest auscultation). One healthy control for each patient, matched by sex, age, and village of residence, was recruited for the study. Nasopharyngeal swabs were collected from participants and tested for 33 pathogens by probe-based multiplex real-time RT-PCR (FastTrack Diagnostics Respiratory pathogen 33 kit). Attributable fraction of illness for a given microorganism was calculated by comparing results between patients and controls (= 100 * [OR − 1]/OR) (OR = odds ratio). Between 24th June 2019 and 24th June 2020, 205 consenting ARI patients and 205 matching controls were recruited. After excluding eight pairs due to age mismatch, 197 pairs were included in the analysis. Males were predominant with sex ratio 1.2:1 and children < 5 years old accounted for 59% of participants. At least one potential pathogen was detected in 173 (88%) patients and 175 (89%) controls. ARI in admitted patients were attributed to influenza B virus, influenza A virus, human metapneumovirus (HMPV), and respiratory syncytial virus (RSV) in 17.8%, 17.2%, 7.5%, and 6.5% of participants, respectively. SARS-CoV-2 was not detected in any cases or controls. Determining ARI aetiology in individual patients remains challenging. Among hospitalised patients with ARI symptoms presenting to a provincial hospital in Northeastern Laos, half were determined to be caused by one of several respiratory viruses, in particular influenza A virus, influenza B virus, HMPV, and RSV.

## Introduction

Acute respiratory infections (ARIs) were classified as the fourth most common cause of death worldwide in 2016, with children under 5 years old being the most affected^1^. Establishing which respiratory pathogens cause ARIs is important for targeting prevention and treatment, avoiding unnecessary use of antibiotics, and developing specific public health strategy^2^. Diagnosing the cause of pneumonia is challenging since few patients are bacteraemic, and sampling the lower respiratory tract is too invasive for routine investigation. Diagnosis frequently relies on sampling of the upper respiratory tract. The advent of molecular techniques permits identification of a broad range of viral and bacterial respiratory microorganisms^3,4^. PCR testing of nasopharyngeal swabs has been shown to be highly sensitive^5^, but the microorganism detected may not necessarily be clinically significant and could simply represent nasopharyngeal carriage. Increasing nasopharyngeal pneumococcal colonization density has been shown to be associated with severe pneumonia in children < 5 years with ARIs^6^; however, this association is still controversial^7^. One approach to aid in causal determination of ARIs is to recruit controls from the same population to characterize the nasopharyngeal microbiome and estimate the fraction of ARI cases attributable to the detected microorganism. The Pneumonia Etiology Research for Child Health (PERCH) study recruited 4232 patients aged < 5 years admitted with severe pneumonia, and 5119 community controls at nine sites in seven low-or middle-income countries (LMICs) in Africa and Asia between 2011 and 2014. Viruses accounted for most pneumonia (61% of 1769 cases) overall, with 31% of all cases attributable to respiratory syncytial virus (RSV)^8^. In the PERCH study, to limit biases in the estimation of pneumonia fraction attributable to the detected microorganism, participants presenting with respiratory symptoms were not excluded from acting as controls, as long as they did not meet the definition of a case^9^.

In Laos, the probability of dying before 5 years of age is 47/1000 live births, and the death rate due to acute lower respiratory infections was estimated as 10/1000 live births in 2017^1^. The 13-valent pneumococcal conjugate vaccine (PCV13) was introduced in late 2013 with limited catch-up vaccination per the national immunization schedule^10^. Post-vaccine observational studies in the capital Vientiane showed a reduction in nasopharyngeal carriage of PCV-13 serotypes following the vaccine’s introduction^11^. In another study of 383 children < 5 years of age hospitalized with confirmed ARI at Mahosot Hospital in Vientiane (2014), the most common virus detected was RSV in 41% of tested children^12^. Several other studies conducted elsewhere in the country have described viral pathogens detected in patients with respiratory infection^13–16^.

The aim of our study was to understand which pathogens potentially caused ARIs in patients at Xiengkhuang Provincial Hospital in Northeastern Laos, and to describe bacteria and viruses colonizing the nasopharynx in controls in the community.

## Methodology

This case-control study was conducted at Xiengkhuang Provincial Hospital (19° 26′ 47″ N, 103° 12′ 11″ E), Laos, from 24th June 2019 to 24th June 2020 with case to control ratio of 1:1.

### Case recruitment

Cases were febrile patients with acute respiratory symptoms admitted to Xiengkhuang Provincial Hospital, and recruited as part of an ongoing larger study into the epidemiology of fever (EFS). Criteria for patient inclusion in EFS were: all patients of any age admitted at hospital with fever (tympanic temperature ≥ 37.5 °C) of any duration, or history of fever in the last 72 h. Participants were screened and recruited from Monday to Friday. Patients were recruited in the current study if they had, in addition to EFS criteria, at least one of the following symptoms: cough, runny nose, nasal congestion, sore throat, difficulty breathing, and/or abnormal chest auscultation. Physical examination was performed by ward doctors and clinical, socio-demographic, medical history, and living environment data were collected using a standardized case record form.

### Control recruitment

After each case recruitment, the study team went to the patient’s village to include a control with similar age (± 1 year for children less than 15 years old, ± 5 years for adult) and same sex as the patient, within two weeks of case enrolment. The matched control could not be selected randomly as initially planned as there were no reliable records about the age of residents. We therefore selected the control who was present at the time when the team went to the village following suggestions given either by cases, cases’ parents, or village authorities.

Controls were recruited if they had no history of hospital admission in the last 30 days, and did not meet the definition of ARI, which meant they did not present concomitant fever and respiratory symptoms but they could have either fever or respiratory symptoms, and if they consented to participate in the study. Physical examination was performed and data collected on a case record form. The controls were followed-up after seven days by phone to see whether they developed fever with respiratory symptoms. If they did, they were excluded from the study. If the date was still within two weeks of case recruitment, another control was recruited.

### Specimen collection and laboratory assays

Nasopharyngeal swabs were collected by the trained study team from cases and controls using flocked swabs (FLOQSwabs^®^ 516CS01, COPAN Diagnostics Inc., Brescia, Italy) which were inserted gently into the nose at an angle parallel to the roof of the mouth about one-half to two-thirds the distance from the nostril to ear lobe and rotated, then the swabs were put into the tube containing virus transport medium, Sigma Virocult^®^ (1 mL, Medical Wire & Equipment, Corsham, UK), then transferred in a cool box to the laboratory of the provincial hospital. On arrival, they were aliquoted into two tubes of 500 μL and kept at − 20 °C before being shipped on dry ice to the Lao-Oxford-Mahosot Hospital-Wellcome Trust Research Unit on a monthly basis. 400 µL of each swab were extracted using EZ1 virus mini kit (Qiagen, Manchester, UK), elution volume of 120 µL, following the manufacturer’s instructions. Then, 10 µL of extract was submitted to multiplex probe based real-time RT-PCR, using the Fast Track Diagnostics Respiratory pathogen 33 kit (Fast-Track Diagnostics, Luxembourg, Luxembourg), following the manufacturer’s instruction for: influenza viruses: A (with identification of H1N1), B, C; human rhinoviruses (HRV); human coronaviruses (HCoV): NL63, 229E, OC43, HKU1; human parainfluenza viruses 1, 2, 3 and 4 (PIV 1–4); human metapneumoviruses (HMPV); human bocaviruses (BocaV); human respiratory syncytial viruses (RSV); adenoviruses (ADV); enteroviruses (EV), human parechoviruses (HPeV); *Mycoplasma pneumoniae*; *Chlamydophila pneumoniae*; *Staphylococcus aureus*; *Streptococcus pneumoniae*; *Haemophilus influenzae*; *Moraxella catarrhalis*; *Klebsiella pneumoniae*; *Legionella* spp.; *Pneumocystis jirovecii*; *Bordetella pertussis*; *Salmonella* spp.. From January 2020 specimens were also tested for SARS-CoV-2 by probe based real-time RT-PCR using E-gene system as described previously^17^. The qRT-PCR assays were considered as positive if the Cq value was < 40.

### Statistical analysis

Socio-demographic characteristics as well as clinical signs and symptoms were reported as proportion or median and interquartile range. Conditional logistic regression reporting odds ratios (ORs), which allows one to take into account matching, was used to assess the association between living environment factors and outcomes which were coded 0 for controls and 1 for ARI cases. Multivariable regression for independent association was not performed as univariate analysis did not show any association apart from ethnicity variable. The same logistic regression was also used to test the association between each binary exposure (organism) and the case–control status. The proportion of individuals found positive by qRT-PCR for a given microorganism was calculated in controls and in patients, and then compared by calculating OR. The probability of the microorganism detected in patient to be the cause of the disease was estimated by calculating the attributable fraction among the exposed (AFE = [OR − 1]/OR)^18^. The attributable fraction (AF), estimation of the proportion of patients attributable to a given microorganism, was calculated by multiplying AFE by prevalence in patients of the given organism. AFE and AF can be calculated only for microorganism with OR > 1. Taking influenza B virus as an example with OR = 18.5 and proportion in patients = 18.8% (Table 2 in “Results” section), AFE = (18.5 − 1)/18.5 = 94.6% and AF = (94.6 × 18.8)/100 = 17.8%. Analysis was performed by using Stata software version 14 (College Station, Texas).

### Ethics

Ethical approval for this study was granted by the Lao National Ethics Committee for Health Research (http://www.laohrp.com/) (NECHR 043, 17th May 2019) and the Oxford Tropical Ethics Committee (OxTREC) (http://www.tropicalmedicine.ox.ac.uk/oxtrec) (OXTREC 027–14, 13th June 2019).

Adult patients, parents, or guardians for participants younger than 15-years-old, provided written informed consent. Assent was obtained for children aged between 12 and 14 years. By chance, no participants aged 15 years old were recruited in the study. Controls or parents or guardians of controls were informed about the objectives of study and asked for written informed consent. The study was performed in accordance with the Declaration of Helsinki and ICH-GCP.

## Results

### Participant description

Between 24th June 2019 and 24th June 2020, among 403 febrile patients recruited in EFS at Xiengkhuang Provincial Hospital in Northeastern Laos, 205 (51%) patients met the definition of ARI and were recruited as cases (Table 1). The same number of controls matched by age, sex, and village of residence to the cases was also recruited with the median time after case recruitment being 9 days (range: 3–14 days). Eight pairs were excluded due to > 1 year age mismatch and none were excluded at seven days of follow up. Therefore, 197 pairs remained in the analysis.

**Table 1:** Participant characteristics and their clinical signs and symptoms.

Variable	Case, n = 197	Control, n = 197
**Characteristics**		
Male:Female	108:89	108:89
Age (year), median (IQR)	4 (0.41–65)	4.1 (0.41–67)
Age group, n (%)		
≤ 2 years old	58 (29.4)	51 (25.9)
> 2 to ≤ 5 years old	58 (29.4)	65 (32.9)
> 5 to ≤ 15 years old	59 (29.9)	59 (29.9)
> 15 years old	22 (11.2)	22 (11.2)
**Symptoms and signs**		
Number of days of fever, median (IQR)	3 (2–5)	0
History of fever over the last 72 h, n (%)	197 (100)	0
Tympanic temperature (ºC), median (IQR)	38.0 (37.2–38.3)	36.9 (36.7–37)
Rigors, n (%)	20 (10.2)	0
Headache, n (%)	57 (28.9)	0
Arthralgia, n (%)	3 (1.5)	1 (0.5)
Myalgia, n (%)	28 (14.2)	2 (1)
Back pain, n (%)	3 (1.5)	1 (0.5)
Nausea, n (%)	34 (17.3)	0
Vomiting, n (%)	68 (33.1)	0
Dysuria, n (%)	3 (1.5)	0
Diarrhea, n (%)	22 (11.2)	0
Abdominal pain, n (%)	9 (4.6)	0
Shortness of breath, n (%)	19 (9.6)	0
Difficulty breathing, n (%)	30 (15.2)	0
Cough, n (%)	167 (84.8)	0
Sputum, n (%)	105 (53.3)	0
Sore throat, n (%)	24 (12.2)	0
Runny nose, n (%)	106 (53.8)	4 (2)
Seizure, n (%)	6 (3)	0
Respiratory rate/min, median (IQR)	29 (20–30)	26 (25–30)
Fast breathing^€^ (for children ≤ 5 years old), n (%)	10/116 (8.6)	0
Abnormal chest auscultation, n (%)	42 (21.3)	0
Pharyngeal erythema, n (%)	143 (72.6)	0
**Patient outcome**		
Pneumonia^$^ (for children ≤ 5 years old), n (%)	10 (8.6%)	0
Severe pneumonia (for children ≤ 5 years old), n (%)	6 (5.2%)	0
Duration of hospitalization (days), median (IQR)	3 (3–5)	0
Death, n (%)	0	0
PCV-13*		
Any PCV vaccination, n (%)	49/100 (49.0)	45/119 (37.8)
1 dose, n	3	4
2 doses, n	1	1
3 doses, n	44	29

^€^Fast breathing = aged 2–11 months: ≥ 50 breaths/min, aged 1–5 years: ≥ 40 breaths/min.

^$^Pneumonia and severe pneumonia were defined according to WHO criteria^19^. Children who presented with cough or difficulty breathing and had fast breathing or chest indrawing, were classified as having pneumonia. Children who presented with cough or difficulty breathing and had at least one of the following criteria were classified as severe pneumonia: oxygen saturation < 90%, while breathing room air, or central cyanosis; severe respiratory distress; signs of pneumonia with a general danger sign (inability to breastfeed or drink, lethargy or reduced level of consciousness, convulsions, vomiting).

*The number of doses were recorded if the vaccination card was seen by the study team. The pneumococcal conjugate vaccine 13 (PCV) was included in the Expanded Program on Immunization in Laos about 5 years before this study was conducted.

A higher proportion of males were recruited in the study (108/197; 54.8%). Children < 15 years old accounted for 175/197 (88.8%) participants, 116/197 (59%) were ≤ 5 years old (Table 1). The main clinical presentations in ARI cases were cough (85%), followed by runny nose (54%), sore throat (12%) and shortness of breath (10%). None of the controls presented either with fever or acute respiratory symptoms (Table 1). Two-thirds of ARI cases were recruited during the rainy season (between May and October). No ARI case was recruited in April 2020 due to the COVID-19 pandemic resulting in travel restrictions and mandatory work-from-home.

The median duration of stay in hospital for patients with ARIs was three days (ranged from 1 to 9 days). Antibiotics were prescribed in 193 ARI patients (98%). Ceftriaxone was the most prescribed in 96 cases (50%), followed by ampicillin in 56 cases (29%), gentamicin in 52 cases (27%), and penicillin in 34 cases (18%). Two or more antibiotics were provided in combination in 59 patients (31%).

### Microorganisms detected

**Figure 1:**
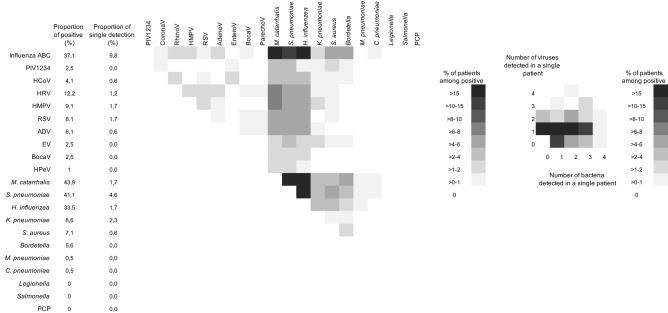
Single and multiple organism detection in ARI cases. PIV, parainfluenza virus; HCoV, human coronavirus; HRV, human rhinovirus; HMPV, human metapneumovirus; RSV, respiratory syncytial virus; ADV, human adenovirus; EV, enterovirus; BocaV, human bocavirus; HPeV, human parechovirus; PCP, *Pneumocystis jirovecii.*

At least one microorganism was detected in 173 (88%) ARI cases. A single organism was detected in 46/173 (27%). Of which, a single bacterial genus was detected in 11% of patients and a single virus in 16%. Influenza virus was the main virus detected alone, in 10% of positive patients. Detection of multiple organisms was common (73%) with a maximum of 6 microorganisms found in four ARI cases (Fig. 1). Amongst the ARI cases with at least one organism detected, influenza virus was commonly co-detected with *M. catarrhalis*, *H. influenzae* and *S. pneumoniae* (20%, 15%, and 14.5%, respectively). The bacteria that co-existed together the most were *M. catarrhalis* and *S. pneumoniae* (co-detected in 30%). More than one virus per patient was found only rarely, with HRV and EV detected in 2.9% (Fig. 1).

The number of patients and controls found positive for each of the 33 microorganisms tested are presented in Table 2. HCoV-HKU1, *L. pneumophila*, and *Salmonella* spp. were not detected in any of the tested samples. PIV3, HPeV, and *M. pneumoniae* were detected in four, two, and one patient, respectively, but not in any controls. PIV2, PIV4, HCoV NL63, and *P. jirovecii* were detected in two, two, three, and one control, respectively, but not in any patients.

**Table 2:** Distribution of microorganisms in patients and controls, detected by qRT-PCR in nasopharynx, odds ratio, AFE, and AF.

Pathogen	Proportion in case, n (%)	Proportion in control, n (%)	OR (95% CI)	AFE, %	AF, %
Influenza viruses	73 (37.1)	3 (1.5)	**35.9 (8.83–146.72)**	97.2	36.1
Influenza B	37 (18.8)	2 (1)	**18.5 (4.45–76.75)**	94.6	17.8
Influenza A	35 (17.7)	1 (0.5)	**35 (4.79–255.47)**	97.1	17.2
Influenza A (H1N1)	9 (4.6)	0	NA	NA	NA
Influenza C	1 (0.5)	1 (0.5)	1 (0.06–15.98)	NA	NA
Parainfluenza viruses	5 (2.5)	5 (2.5)	1 (0.29–3.45)	NA	NA
PIV 3	4 (2)	0	NA	NA	NA
PIV 1	1 (0.5)	1 (0.5)	1 (0.06–15.98)	NA	NA
PIV 4	0	2 (1)	NA	NA	NA
PIV 2	0	2 (1)	NA	NA	NA
Human coronaviruses	8 (4.1)	5 (2.5)	1.75 (0.51–5.97)	42.9	6.3
HCoV 229E	4 (2)	1 (0.5)	4 (0.44–35.78)	75.0	1.5
HCoV OC43	4 (2)	1 (0.5)	4 (0.44–35.78)	75.0	1.5
HCoV NL63	0	3 (1.5)	NA	NA	NA
HCoV HKU1	0	0	NA	NA	NA
HRV	24 (12.2)	55 (31.5)	**0.4 (0.24–0.67)**	NA	NA
HMPV A&B	18 (9.1)	4 (2)	**5.6 (1.66–19.33)**	82.1	7.5
RSV A&B	16 (8.1)	4 (2)	**5 (1.44–17.27)**	80.0	6.5
ADV	12 (6,1)	24 (12.2)	**0.45 (0.21–0.96)**	NA	NA
EV	5 (2.5)	8 (4.1)	0.57 (0.17–1.95)	NA	NA
BocaV	5 (2.5)	8 (4.1)	0.5 (0.12–1.99)	NA	NA
HPeV	2 (1)	0	NA	NA	NA
*M. catarrhalis*	86 (43.6)	123 (62.4)	**0.4 (0.22–0.60)**	NA	NA
*S. pneumoniae*	81 (41.1)	120 (61)	**0.4 (0.27–0.65)**	NA	NA
*H. influenzae*	66 (33.5)	94 (47.7)	**0.51 (0.33–0.79)**	NA	NA
*K. pneumoniae*	17 (8.6)	34 (17.3)	**0.43 (0.23–0.83)**	NA	NA
*S. aureus*	14 (7.1)	47 (23.9)	**0.19 (0.09–0.42)**	NA	NA
*Bordetella* spp.	11 (5.6)	6 (3)	2 (0.68–5.85)	50	2.8
*M. pneumoniae*	1 (0.5)	0	NA	NA	NA
*C. pneumoniae*	1 (0.5)	4 (2)	0.25 (0.03–2.24)	NA	NA
*H. influenzae* B	0	3 (1.5)	NA	NA	NA
*L. pneumophila*	0	0	NA	NA	NA
*Salmonella* spp.	0	0	NA	NA	NA
*P. jirovecii*	0	1 (0.5)	NA	NA	NA

AFE, attributable fraction among the exposed = (1 − 1/OR) * 100; AF, attributable fraction = AFE * Proportion of a given organism in case/100; OR, odds ratio; CI, confident interval; NA, not applicable; HRV, human rhinovirus; HMPV, human metapneumovirus; RSV, respiratory syncytial virus; ADV, adenovirus; EV, enterovirus; BocaV, human bocavirus; HPeV, human parechovirus.

Statistically significant OR are written in bold.

**Figure 2:**
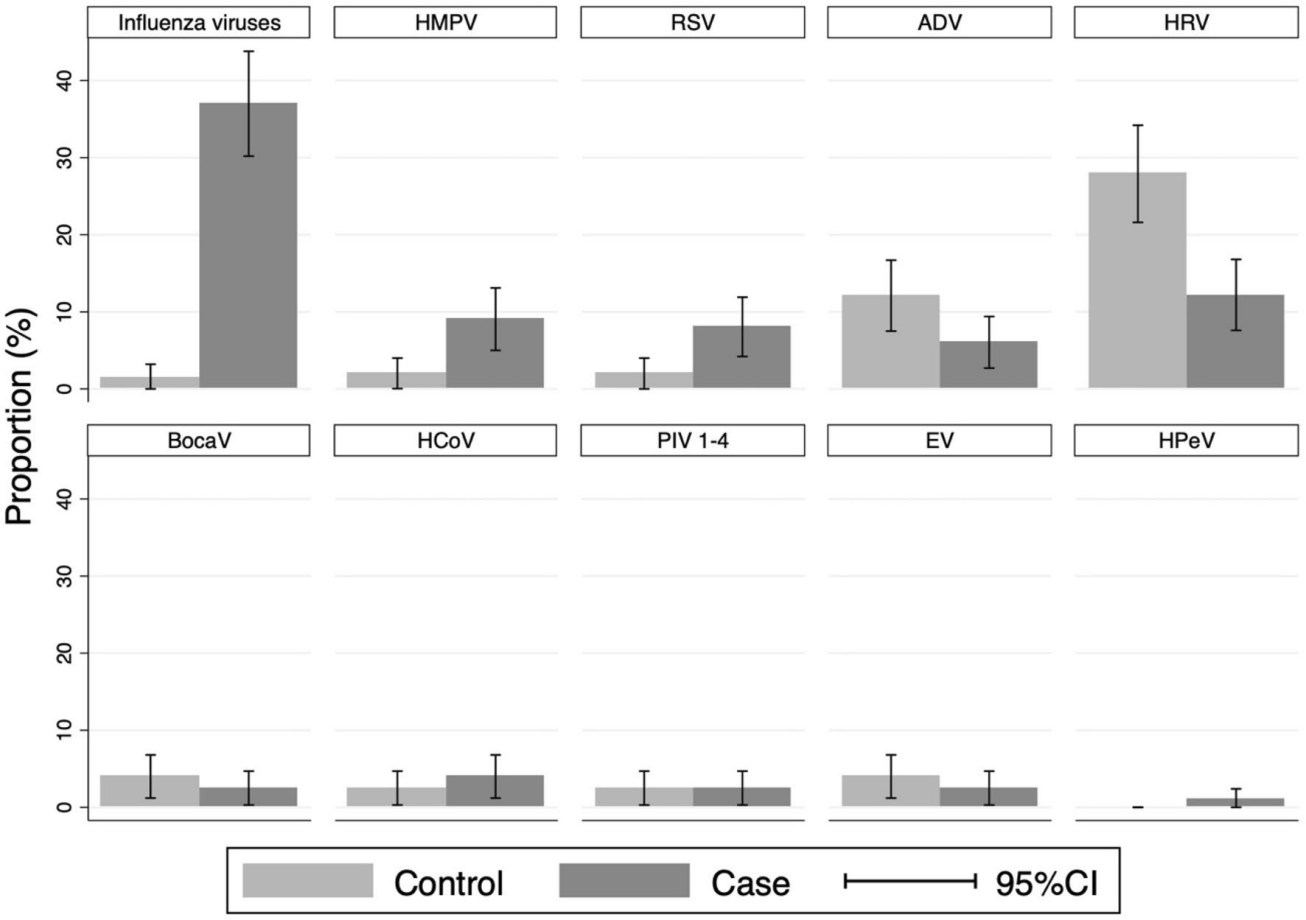
Prevalence of viral pathogens detected among cases and controls. Influenza viruses: influenza A virus, influenza B virus and influenza C virus. HMPV, human metapneumovirus; RSV, respiratory syncytial virus; ADV, adenovirus; HRV, human rhinovirus; BocaV, human bocavirus; HCoV, human coronavirus including HCoV 229E and HCoV OC43, and HCoV NL63; PIV 1–4, parainfluenza virus 1, 2, 3 and 4; EV, enterovirus; HPeV, human parechovirus.

**Figure 3:**
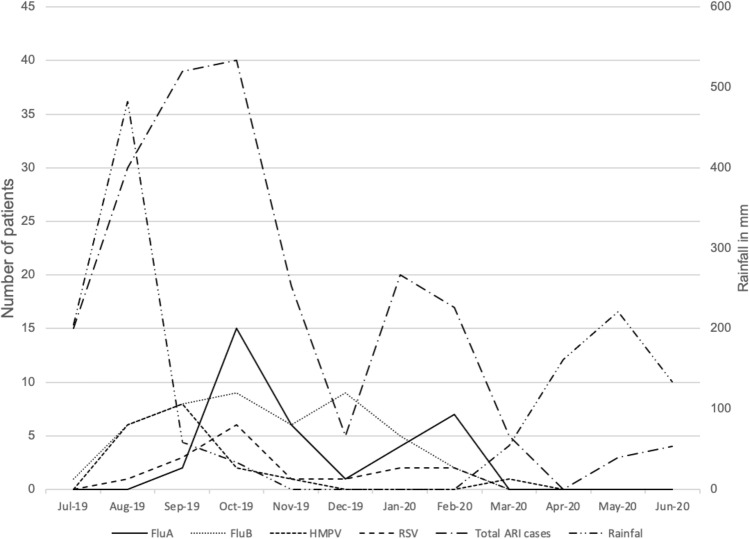
Temporal distribution of detection of influenza A virus, influenza B virus, human metapneumovirus, and respiratory syncytial virus in included ARI patients in relation to monthly average rainfall. Xiengkhuang rainfall data from Department of Meteorology and Hydrology, Lao PDR.

**Figure 4:**
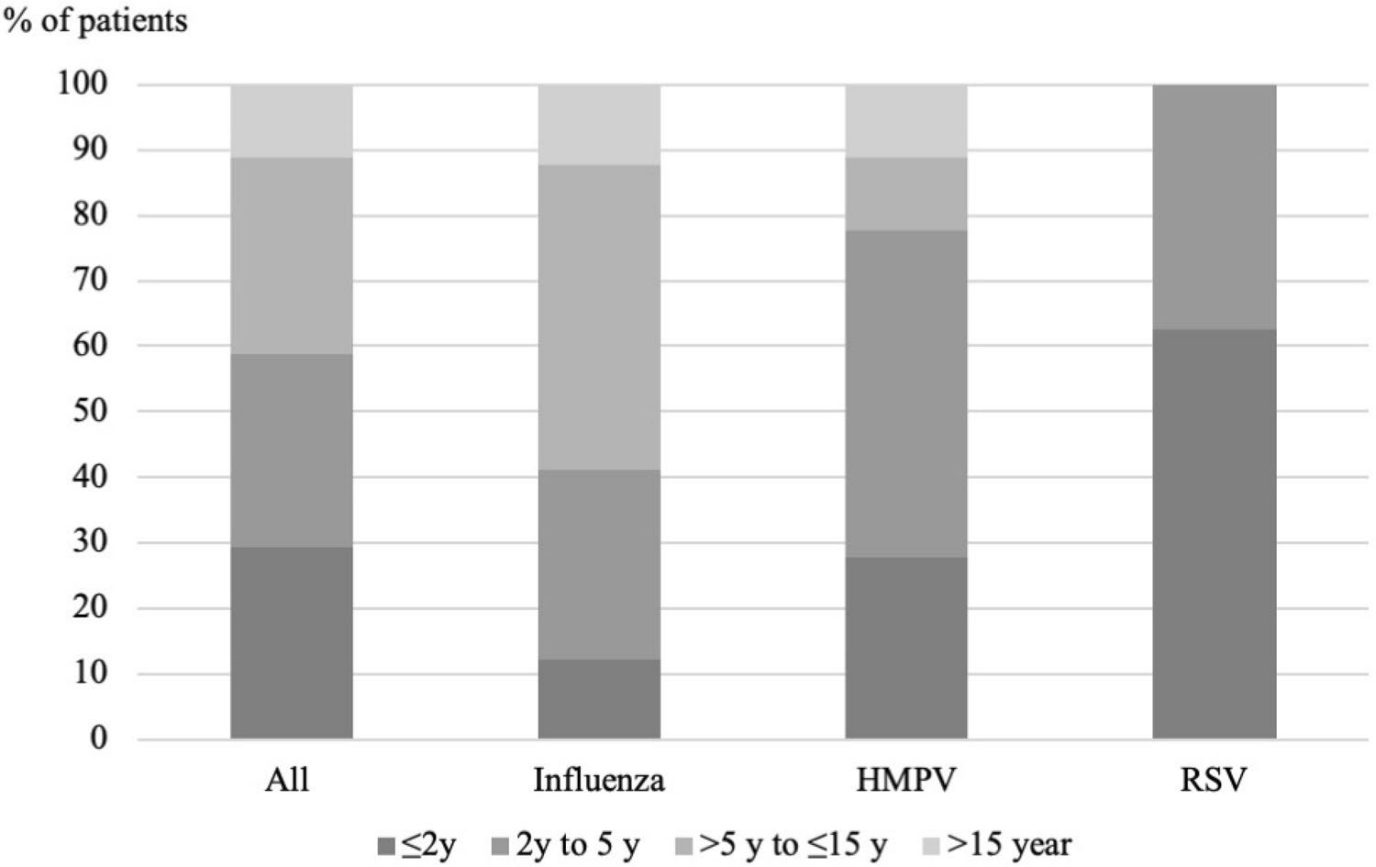
Age distribution in all ARI patients and in patients positive for influenza viruses, respiratory syncytial virus (RSV) and human metapneumovirus (HMPV).

ARIs in patients admitted to Xiengkhuang Provincial Hospital were attributed to influenza A virus, influenza B virus, HMPV, and RSV in 17.2%, 17.8%, 7.5%, and 6.5% patients, respectively. The majority of cases who tested positive for those microorganisms were attributable to them, 97.1%, 94.6%, 82.1%, and 80%, respectively (Table 2 and Fig. 2). We also found that those four microorganisms were more frequently detected during the rainy season than during the rest of the year; 62.5% of RSV positive cases were detected between May and October, 57.5% of influenza virus positive cases, and 83.3% of HMPV positive cases (Fig. 3). RSV was detected only in children < 5 years old, and 62.5% of RSV positive patients were 2-years-old or younger (Fig. 4). Other microorganisms frequently detected in patients were *M. catarrhalis* (43.9%), *S. pneumoniae* (41.1%), *H. influenzae* (33.5%), HRV (12.2%), *K. pneumoniae* (8.6%) and *S. aureus* (7.1%), and ADV (6.1%); however, they were significantly more frequently detected in controls (Table 2). A total of 102 samples from cases and controls recruited since January 2020 were also tested for SARS-CoV-2, and all tested negative.

### Risk factors for ARI

Data on environmental factors collected in patients and controls are shown in Table 3. There were no variables considered as risk factors for ARI in univariate analysis apart from ethnicity. Being Lao Loum (lowland Lao) was significantly (*p* = 0.009) positively associated with ARIs presentation compared to other ethnic groups with OR (95% CI) being 2.77 (1.29–5.95) (Table 3).

**Table 3:** Environmental factors associated with ARI.

Environment	Case, n (%)	Control, n (%)	Univariate analysis	
			Crude OR (95% CI)	*p* value
Lao Loum ethnicity	125 (63.5)	109 (55.3)	**2.77 (1.29–5.95)**	**0.009**
Having children < 5 years in the house	142 (72.1)	148 (75.1)	0.78 (0.44–1.37)	0.397
Crowding^a^	34 (17.3)	34 (17.3)	1 (0.58–1.70)	1
Smoker in the house	56 (28.6)	63 (32.1)	0.84 (0.53–1.34)	0.48
Self-sufficient^b^	191 (96.9)	193 (97.9)	0.5 (0.09–2.72)	0.423
Attended university^c^	103 (52.3)	88 (44.7)	1.48 (0.94–2.33)	0.089
Biomass fuels use^d^	187 (94.9)	177 (89.8)	2.25 (0.97–5.17)	0.05
Indoor pollution^e^	90 (45.7)	87 (44.1)	1.07 (0.69–1.65)	0.74
Untreated drinking water	4 (2)	2 (1)	3 (0.31–28.8)	0.34
No toilet/latrine	1 (0.5)	0	NA	1

Significant values are in bold.

^a^More than 7 people living in the same house.

^b^The income of the family is sufficient to live on judged by family.

^c^Highest form of education in the family.

^d^Charcoal or wood were used in cooking.

^e^Making fire inside the house.

### Pneumonia

Ten of 116 (8.6%) children (≤ 5 year old) patients met the WHO definition of pneumonia Table 1^19^. Two of those had completed PCV-13 vaccination. The microorganisms detected in the nasopharynx of those patients are shown in Table 4. Six children (5.2%) presented with severe pneumonia, one presented with seizure, two with severe respiratory distress, and three with vomiting.

**Table 4:** Organisms detected in nasopharynx in children who met the WHO pneumonia definition^19^.

Patient #	Severe	Flu	HCoV	HRV	HMPV	BocaV	RSV	HPeV	*S. pneu*	*K. pneu*	*M. catarrhalis*	*B. pertussis*	*H. inf*
1	0	0	0	0	0	0	0	0	0	1	0	0	0
2	1	0	0	0	0	0	0	0	1	0	1	1	1
3	1	0	0	0	0	0	0	0	0	0	0	0	1
4	1	0	0	1	1	0	0	0	0	0	0	0	0
5*	1	0	0	0	0	0	1	0	1	0	1	0	1
6	0	1	0	0	0	0	0	0	1	0	1	0	1
7	1	0	0	0	0	0	1	1	1	0	1	0	0
8*	0	1	1	0	0	0	0	0	0	0	1	0	1
9	0	1	0	0	0	0	0	0	1	0	1	0	0
10	1	0	0	0	0	1	0	0	1	0	1	0	1

*Patients have received three doses of PCV-13.

Flu, influenza viruses; HCoV, human coronaviruses; HRV, human rhinovirus; HMPV, human metapneumovirus; BocaV, human bocavirus; RSV, respiratory syncytial virus; HPeV, human parechovirus; *S. pneu*, *Streptococcus pneumoniae*; *K. pneu*, *Klebsiella pneumoniae*; *M. catarrhalis*, *Moraxella catarrhalis*; *B. pertussis*, *Bordetella pertussis*; *H. inf*, *Haemophilus influenzae.*

## Discussion

Our study showed that half of the febrile patients admitted to Xiengkhuang Provincial Hospital between June 2019 and June 2020 and recruited to the EFS study presented with signs and/or symptoms of acute respiratory infections, and the majority of them were children aged less than 15 years old (89%); 59% were aged less than five years. Sixty-six percent (66%) were recruited between May and October. All patients were discharged alive. No risk factor for ARI presentation could be identified. Among patients ≤ 5 years old, 9% (10/116) had pneumonia by WHO criteria, and six of them (60%) had severe pneumonia. The ARIs were attributable to influenza in 36% of cases, human metapneumovirus in 7.5% and respiratory syncytial virus in 6.5% cases. These findings are in accordance with similar studies in literature as reported in the metanalysis from Shi et al. showing that RSV, influenza virus and HMPV were strongly associated with ALRI (clinical pneumonia including bronchiolitis) in children less than five years old^20^. Feikin et al. using a similar approach, in an investigation of nasopharyngeal samples from 766 ARI cases and 273 healthy controls in Kenya, 2007–2010, showed that influenza A virus was the most common virus (22%) and *S. pneumoniae* the most common bacteria (16%) detected in patients^21^. They also showed that influenza virus, RSV and HMPV were associated with illness whereas ADV, parainfluenza viruses, HRV/EV, HPeV, and *M. pneumoniae* were not. In a study conducted in Vietnam, 2007–2010, on 1992 ARI patients and 350 healthy children. HRV (24%), RSV (20%) and influenza A virus (12%) were the main viruses detected in ARI cases. RSV and influenza A virus were associated with increased risk of hospitalisation^22^. In rural Thailand, 2005–2010, among 7388 hospitalized children with acute lower respiratory tract infections (ALRI), the main viruses detected were RSV (20%), HRV (18.7%), bocavirus (12.8%) and influenza viruses (8%). The inclusion of 667 controls permitted to show that RSV, influenza, ADV, HMPV and parainfluenza viruses 1 and 3 were associated with ALRI^23^. Similarly, the PERCH multi-country case–control study with the aim to find the causes of severe pneumonia in children found the same respiratory viruses were commonly associated with the cases. Additionally, *S. pneumoniae and H. influenzae type B* were identified as causal and more common in severe pneumonia^8^. Despite the fact the viruses were likely the predominant cause of ARI, nearly all (98%) of patients were treated with antibiotics. As the results show, by June 2020 Laos had avoided widespread community transmission of SARS-CoV-2 with no cases detected in this study. Evidence from other countries suggests that SARS-CoV-2 has displaced other respiratory viruses that usually circulate, with physical distancing and other lockdown measures thought to have acted to reduce transmission^24,25^.

Although microorganisms were detected in nearly 90% of patients, their potential aetiological role could not be established for most of them. Many respiratory viruses known to cause ARIs were detected in our study, there was no evidence of association of detection of parainfluenza viruses, enterovirus, bocavirus, coronavirus with acute respiratory illness. Moreover, it is noted that adenovirus, and rhinovirus were detected more frequently in controls who had no history of ARIs within the last 14 days before recruitment and did not develop any respiratory symptoms seven days after recruitment based on the report of participants or family. The fact that those microorganisms were found more frequently in healthy individuals does not mean that those microorganisms were definitely not the cause of ARI in some cases.

The nasopharynx is colonized by a variety of different microbial species. Most research focuses on bacteria, and the role of viruses in the composition of the respiratory microbiota is likely to be underestimated^26^. *Moraxella catarrhalis*, *Staphylococcus aureus*, *Streptococcus pneumoniae*, *Klebsiella pneumoniae*, and *Haemophilus* spp., which were found in our study associated with controls, are well known frequent contributors to the respiratory microbiota. In the PERCH study, *S. pneumoniae* was, as in our study, significantly more frequently detected in controls than in patients^8^. However, Ho Man et al. showed in a matched case–control study in a paediatric intensive care unit that *S. pneumoniae* and *H. influenzae* were strongly associated with lower respiratory tract infection^27^. The composition of the upper respiratory tract is dynamic with lifelong evolution of organism diversity and quantity. Recent interest in studying the role of the microbiome has provided evidence on the complexity of the factors influencing microbiome changes, and of the role that the microbiome could have in development of disease. Schippa et al. found that there was a shift in the content of nasal microbiota with significantly lower biodiversity in children with bronchiolitis due to RSV^28^. In our study, antibiotic treatment, as well as viral infection, in ARI patients may have caused changes in the microbiome. However, Ramos-Sevillano et al. found only minor perturbation of the nasopharyngeal microbiome during influenza infection^29^. Although, *Moraxella catarrhalis*, *Staphylococcus aureus*, *Streptococcus pneumoniae*, and *Klebsiella pneumoniae* are reported as causes of community acquired pneumonia, their presence in samples collected from the upper respiratory tract is difficult to interpret. Over the last two decades, the introduction of vaccines against *Haemophilus influenzae* type B and *S. pneumoniae* have led to a substantial reduction in incidence and severity of childhood pneumonia^30^. In Laos, the PCV 13 coverage is heterogeneous. The median PCV 13 coverage increased from 4.5% in 2014 to 37.5% in 2017 among children less than five years and the PCV 13 coverage had indirect effects on pneumococcal vaccine-type carriage^10^.

This study highlights the difficulty in making a definitive etiological diagnosis of ARI. The technological breakthrough of molecular assays, more specifically probe based real-time PCR, in the past decades have greatly helped to understand the diversity of pathogens causing respiratory tract illness^31,32^. The development of syndromic commercial kits, as FTD or Film Array, permit easy implementation of microorganism screening in diagnostic laboratories. The counterpart of highly sensitive detection of microorganism using real-time PCR is a decrease in specificity due to the detection of microorganisms naturally carried in upper respiratory tract at low density (URT). Conventionally, pneumonia aetiology is identified by detecting microorganisms in lung or in normally sterile body fluids such as blood. However, investigation of lung tissue is invasive and usually not available, and detection of microorganisms in blood has low sensitivity. Hammit et al., discussed these challenges in determining the aetiology of pneumonia and some strategies to overcome them^18^. The case–control study approach permits estimation of the attributable fraction for each tested microorganism, facilitating the identification of the main aetiologies at the population level. However, this does not permit to establish the aetiology at individual level. Indeed, the attributable fraction only provides a probability for the detected microorganism to be the cause of the disease. Moreover, the attributable fraction cannot be calculated for the microorganisms which are more frequently detected in controls than in cases, whereas their causal role cannot be excluded. In addition, our findings show that this strategy is not applicable for many respiratory pathogens which are part of the normal commensal flora. In the PERCH study, an integrated analysis method using a Bayesian approach was used. This method integrated results for multiple tests and permitted estimation of aetiology at individual level for a large panel of microorganisms. This approach requires the use of a combination of various tests from different samples, and the assumption for key parameters, such as aetiology fraction, sensitivity of all tests used and uncertainty range. This is difficult to implement in low-income countries where sampling from lower respiratory tract is rarely performed.

Microorganism detection in URT showed low specificity for most microorganisms- only influenza A virus, influenza B virus, metapneumovirus and RSV had an attributable fraction among the exposed of over 80%. In term of diagnosis, this means that the positive predictive value of the detection of microorganisms in the URT is elevated for HRSV, HMPV and influenza virus.

Seasonal influenza, a common cause of acute respiratory illness, is usually self-limiting in healthy people. However, younger children and elderly people are at risk of severe disease with secondary bacterial infection resulting from perturbation of the microbiome in the upper respiratory tract and the translocation of colonized bacteria to the lower respiratory tract^33,34^. In our study, 30 (40%) of influenza positive patients were less than five-years-old, and three of them did not have severe pneumonia. RSV is the number one cause of lower respiratory tract infection in young children, frequently causing bronchiolitis and pneumonia. In our study, there were only ten children aged less than five years who met the WHO pneumonia definition and RSV was found in two of them. However, all were judged to require hospitalisation by the assessing clinicians. HMPV is also an important cause of respiratory infection in children, causing spectrum of illness from mild disease to bronchiolitis and pneumonia. HMPV infection is usually less severe than influenza virus and RSV infections^35^. In our study, only one child with severe pneumonia had HMPV infection.

Our study has important limitations. First, the sample size was small- a larger sample size with severe disease might give better understanding of the roles of these organisms and permit analysis stratified by age. Second, we could not determine whether some detected organisms were the causes of acute respiratory infection based only on nasopharyngeal swab and AFE since those organisms were more commonly found in healthy controls. Third, the number of patients with ARI presented to hospital was not representative of the true epidemiology due to the COVID-19 pandemic resulting in travel restrictions, patients fear of coming to hospital during the second 6 months of the study. The first COVID-19 control measures in Laos were implemented quite early in February 2020 by national recommendation for wearing face masks, hand hygiene and respiratory etiquette. The first COVID-19 case was reported on March 24, 2020, leading the government to strengthen the measures with suspension of internal and international travels followed by a nationwide lockdown from 1st April to 3rd May 2020^36^. Finally, some ARI patients who were admitted during the weekend were not included to the study. This might cause selection bias. However, patients admitted during the weekend were in general not many compared to work days and patients admitted on Sunday could be recruited on Monday if they met the inclusion criteria.

In conclusion, ascertaining which pathogens detected from nasopharyngeal swabs are the cause of ARI is still challenging and complex due to the limitation of analytic methods. By using the AFE estimate, we showed the importance of influenza A virus, influenza B virus, human metapneumovirus, and respiratory syncytial virus infections as causes of ARI in hospitalized patients in Northeastern Laos. This is supported by data from other studies and suggests that a modified testing approach targeting these organisms might be useful to facilitate appropriate antibiotic use and for surveillance in Laos. URT swabbing is an easy and reliable technique to identify cases caused by those viruses, enabling implementation of extended surveillance which would permit to better understand the burden of those infections at country level.

## Data Availability

The data are available upon request to the Mahidol Oxford Tropical Medicine Research Unit Data Access Committee (http://www.tropmedres.ac/data-sharing) for researchers and following the Mahidol Oxford Tropical Medicine Research Unit data access policy (http://www.tropmedres.ac/_asset/file/datasharing-policy-v1-1.pdf). Queries and applications for datasets should be directed to koukeo@tropmedres.ac.
